# Repetitive transcranial magnetic stimulation for post-stroke depression: An overview of systematic reviews

**DOI:** 10.3389/fneur.2023.930558

**Published:** 2023-03-16

**Authors:** Wanning Gao, Fuyu Xue, Bin Yu, Shuo Yu, Weimin Zhang, Haipeng Huang

**Affiliations:** ^1^School of Rehabilitation Medicine, School of Integrated Traditional Chinese and Western Medicine, Changchun University of Chinese Medicine, Changchun, China; ^2^Acupuncture and Tuina Department, The Third Affiliated Hospital of Changchun University of Chinese Medicine, Changchun, China; ^3^School of Traditional Chinese Medicine, Changchun University of Chinese Medicine, Changchun, China; ^4^College of Acupuncture and Tuina, Changchun University of Chinese Medicine, Changchun, China; ^5^Encephalopathy Department, The Third Affiliated Hospital of Changchun University of Chinese Medicine, Changchun, China; ^6^Research Institute of Acupuncture and Tuina, Changchun University of Chinese Medicine, Changchun, China

**Keywords:** repetitive transcranial magnetic stimulation, post-stroke depression, stroke, overview, non-invasive brain stimulation

## Abstract

**Objective:**

There is conflicting published research about the clinical effectiveness of repetitive transcranial magnetic stimulation (rTMS) for the treatment of post-stroke depression (PSD). In order to provide trustworthy information for upcoming therapeutic treatments, this review attempts to compile and assess the data from pertinent systematic reviews and meta-analyses.

**Methods:**

The systematic evaluation of repetitive transcranial magnetic stimulation for post-stroke depression was collected by searching CNKI, VIP, Wanfang Database, CBM, PubMed, EMBASE, Web of Science, and Cochrane Library. The retrieval time is from database construction to September 2022. After selection, the included literature was evaluated for methodological quality, reporting quality, and evidence quality using AMSTAR2, PRISMA statements, and the GRADE system.

**Results:**

There were a total of 13 studies included, with three having generally comprehensive reporting according to the PRISMA statement, eight having some reporting issues, two having pretty substantial information issues, and 13 having extremely poor methodological quality according to the AMSTAR2. The GRADE was used to grade the quality of the evidence, and the included literature had 0 high-level evidence, eight medium-level evidence, 12 low-level evidence, and 22 very low-level evidence.

**Limitations:**

The results of this study are from researchers' subjective evaluation and only qualitative analysis, not quantitative evaluation. Although repeated cross-evaluation of researchers is carried out, the results will be personal. The interventions included in the study were complex, and it was impossible to analyze their effect values quantitatively.

**Conclusion:**

Patients with post-stroke depression may benefit from repetitive transcranial magnetic stimulation. However, in terms of the quality of the reports, the methodology, and the quality of the evidence, published systematic evaluations/meta-analyses are of low quality. We list the drawbacks of the current clinical trials of repetitive transcranial magnetic stimulation for post-stroke depression as well as potential therapeutic mechanisms. This information may serve as a guide for future clinical trials aiming to establish a solid foundation for the clinical efficacy of repetitive transcranial magnetic stimulation in the treatment of post-stroke depression.

## Highlights

- This paper fills a gap in the literature by providing the first overview of systematic reviews of repetitive transcranial magnetic stimulation (rTMS) for post-stroke depression (PSD).
- There were a total of 13 studies included, with three having generally comprehensive reporting according to the PRISMA statement, eight having some reporting issues, two having pretty substantial information issues, and 13 having extremely poor methodological quality according to the AMSTAR2. The GRADE was used to grade the quality of the evidence, and the included literature had 0 high-level evidence, eight medium-level evidence, 12 low-level evidence, and 22 very low-level evidence.
- The evidence that is currently available points to rTMS's efficacy in the treatment of PSD. Presently, systematic reviews and meta-analyses of rTMS for the treatment of PSD have poor methodological quality, poor reporting quality, and scant empirical support.
- The findings of this study may have therapeutic relevance and some translational value. This review aims to summarize and assess the evidence from pertinent systematic reviews and meta-analyses, to highlight the limitations of rTMS for PSD in current clinical practice, to summarize the potential mechanisms of rTMS for PSD, to provide a direction for future clinical studies, and to propose that future studies should strictly follow the recommendations made in this review. The study also recommended that future research strictly adhere to the rules of scientific research design in order to strengthen the validity of the findings and offer a high-quality foundation for enhancing the clinical effectiveness of rTMS for PSD.

## 1. Introduction

One of the most frequent neuropsychiatric complications following a stroke is post-stroke depression (PSD), which includes depressive symptoms and corresponding somatic symptoms. According to the DSM V, depressive disorder is characterized by the presence of a depressed, empty, or irritable mood along with somatic and cognitive changes that have a significant impact on a person's ability to function. In the immediate aftermath of a stroke, the prevalence of PSD can reach 40%, and after 5 years, it can reach 31% (1, 2). PSD has been demonstrated to have an impact on brain function in recent studies. Depressive symptoms may hasten the onset of cognitive dysfunction and exacerbate cognitive dysfunction, seriously impeding stroke patients' recovery, decreasing the effectiveness of rehabilitation, raising the risk of disability, death, and stroke recurrence, and placing a significant psychological and financial burden on families and society (3–5).

Currently, Western medicine mostly uses medication, physical therapy, and psychotherapy for the therapeutic treatment of PSD. Antidepressants, such as tetracyclic antidepressants, tricyclic antidepressants, and others, are still the most popular option among them. Psychotherapy includes behavioral therapy, family therapy, etc. Electroconvulsive therapy, hyperbaric oxygen therapy, and other techniques are used in physical therapy (6). On the other hand, traditional medical procedures like acupuncture, moxibustion, and tui na are frequently used in Chinese medicine. However, it takes at least 3–4 weeks for medicine to produce a clinical response, and only 50% of patients experience remission (7, 8). The use of numerous types of antidepressants not only raises the risk of stroke recurrence but also has contraindications for usage when mixed with some stroke drugs (9), some patients have poor long-term medication compliance and even severe responses. The effectiveness of psychotherapy and other treatments is also impacted by somatic dysfunction and cognitive dysfunction in stroke patients as a result of neurological abnormalities, which to some extent restricts the use of the aforementioned treatment options in clinical practice. For these reasons, attention has been focused on non-pharmacological treatments for depression. In recent years, moderate daily coffee consumption has been found to be significantly associated with a reduced risk of depression (10), and several systematic reviews and Meta-analyses (11–13) have shown the beneficial effect of caffeine on improving cognitive decline in depressed patients, especially for non-smokers (14). Transcranial magnetic stimulation (TMS), another non-pharmacological treatment, has attracted a lot of interest.

TMS is a non-invasive physical therapeutic method that simulates neuronal excitation or inhibition in the brain for prolonged programmed stimulation in order to produce neuromodulatory effects on the cerebral cortex. Specific neural pathways produced by the cerebral cortex are modulated by TMS, and different stimulation frequencies have distinct impacts on neuronal activity. Low-frequency TMS was reported to produce inter-synaptic long-term depression in human brain slices. As opposed to this, high-frequency TMS caused long-term potentiation, a crucial sign of functional synaptic plasticity and the main mechanism by which TMS affects functional neural plasticity to treat a range of disorders.

Current, the effectiveness of TMS for dementia and vascular cognitive diseases has gained more and more attention, and numerous studies have documented numerous ongoing clinical benefits from its use, including improved auditory comprehension, behavior, recognition, and memory (15, 16). The FDA has approved transcranial magnetic stimulation (TMS) as a non-invasive treatment for depression, and the 2014 Evidence-Based Medicine Guidelines gave TMS an A recommendation for the treatment of depression. The clinical TMS society consensus review and treatment recommendations for TMS therapy for major depressive disorder recommends TMS therapy can be administered with the concomitant administration of antidepressants or other psychotropic medications (17). Transcranial magnetic stimulation has also been demonstrated to be effective in the treatment of depression. Numerous studies have demonstrated the efficacy and safety of TMS for Major depressive disorder patients (17, 18).

Repetitive transcranial magnetic stimulation (rTMS) for PSD has been the subject of evidence-based medical research in recent years, and various original investigations and systematic reviews (SR) pertaining to rTMS for PSD have been published. However, there is variability in the outcome indicators of the individual studies or systematic evaluations that have been published, and the quality of the articles varies. It has been demonstrated that high-quality systematic reviews offer the best clinical recommendations for evidence-based medicine, whereas low-quality systematic reviews may mislead decision-makers. Reassessing the existing systematic reviews, meta-analyses, and systematic evaluations of the literature on rTMS for PSD is crucial. A thorough, systematic analysis of the data supporting the efficacy of rTMS for PSD has not yet been published.

The overview of systematic review is a complete research strategy that involves collecting and re-evaluating all pertinent systematic evaluations of a disease's prognosis, diagnosis, and treatment. Because it incorporates the results of systematic evaluation more thoroughly and contains more information, it offers higher quality evidence for clinical action (19). In order to ensure that scientific evidence with little bias and a tendency to be true is used in clinical practice, it has been suggested that systematic evaluation should have strong quality control procedures (20). There are many available methodological tools and reporting criteria (21). For analyzing systematic evaluations of intervention studies, including randomized controlled trials and non-randomized controlled trials, AMSTAR2 is regarded as being very reliable and useful (39). To further assist writers in better drafting and reporting of systematic evaluations/Meta-analyses, the PRISMA statement offers a revised summary based on QUOROM. This demonstrates the 16 AMSTAR 2 items and the 27 PRISMA items, some of which are complementary to one another. PRISMA places more emphasis on the organization of systematic assessment, whereas AMSTAR 2 is more concerned with the specifics of the initial investigation of systematic evaluation. For instance, while AMSTAR 2 emphasizes funding sources in the original study, item 27 of the PRISMA statement emphasizes the role of funding sources and other support for systematic evaluation. Both quality indicators take into account potential conflicts of interest, but only AMSTAR 2 specifically lists conflict-of-interest items. The combined use of the two allows for a comprehensive assessment of the methodological quality of the systematic evaluation and the quality of the report quality.

As a result, this paper aims to evaluate the clinical efficacy, methodological quality, report quality, and level of evidence of the published systematic reviews, meta-analyses, and systematic evaluations involving rTMS for PSD using the PRISMA statement, AMSTAR2 scale, and GRADE system. This will allow us to systematically and thoroughly compare the effectiveness of repetitive rTMS to other therapies for PSD, in order to provide a basis for clinical treatment.

## 2. Data and methods

### 2.1. Inclusion and exclusion criteria

#### 2.1.1. Study type

Published systematic evaluation/Meta-analysis/systematic review of the literature on repetitive transcranial magnetic stimulation for post-stroke depression, regardless of study geography, limited to Chinese and English.

#### 2.1.2. Subjects

Post-stroke depression patients meeting diagnostic criteria, regardless of gender, age, race, time of onset, or duration of illness.

#### 2.1.3. Interventions

The test group was repeated transcranial magnetic stimulation with unlimited frequency, treatment site and other treatment parameters, or duplicated transcranial magnetic stimulation combined with other treatments, including antidepressants, acupuncture therapy, rehabilitation therapy and conventional therapy for cerebrovascular disease; the control group was treated with drugs, sham stimulation and other medicines or blank control, and the control group should be consistent with the baseline of the treatment group.

#### 2.1.4. Outcome indicators

① Outcome indicators for effective clinical efficacy: improvement of depressive symptoms including one or more of the commonly used depression evaluation scales such as Hamilton Depression Scale (HAMD), Montgomery and Asperger Depression Rating Scale (MADRS) score, Beck Depression Self-Rating Scale (Beck); improvement of neurological function including NIHSS score, etc.; improvement of cognitive function including Montreal Cognitive Assessment (MOCA), Concise Mental State Scale (MMSE), etc.; improvement of daily living ability including Barthel Index (BI), Modified Barthel Index (MBI), etc.; cure rate, efficiency rate. ② Safety: adverse reactions, etc.

#### 2.1.5. Exclusion criteria

① Duplicate published literature. ② Dissertation or conference papers. ③ Literature comparisons were made between two duplicate transcranial magnetic stimulation methods. ④ Incomplete information or incorrect data in the literature.

### 2.2. Retrieval strategy

Computer searches of PubMed, EMBASE, Cochrane Library, Web of Science, China Knowledge Network (CNKI), VIP, Wanfang Database, and China Biomedical Literature Database (CBM) were conducted to collect systematic evaluations of repeated transcranial magnetic stimulation for post-stroke depression; all searched over the time frame from database creation to September 2022. The search terms included depression, depressed, post-stroke depression, stroke, brain vascular accident, non-invasive brain stimulation, repetitive transcranial magnetic stimulation, transcranial magnetic stimulation, meta-analysis, systematic assessment, systematic review, systematic evaluation, system evaluation, and systematical review. In addition, we manually searched for reviews related to the repetitive transcranial magnetic stimulation and post-stroke depression, ongoing clinical trials, and unpublished conference papers and dissertations. We used Boolean logic to formulate the retrieval formula, which was applicable to all databases. The PubMed searching strategy is included in Appendix A, and the other search strategies are available in Appendix B.

### 2.3. Literature screening and data extraction

The systematic evaluation/meta-analysis/systematic review literature obtained from the search was imported into Endnote, duplicates were removed, and two researchers (WN-G and FY-X) independently screened the titles and abstracts to select potentially helpful literature. The full article of potentially valuable studies was obtained. Two researchers read the entire article independently and made the final decision. The screening was done by first reading the title and abstract. After excluding irrelevant literature, the whole piece was further read to exclude literature that did not meet the inclusion and exclusion criteria. Data were extracted from the included literature, and the information extracted included: author, country, patient age, study type, literature size, sample size, treatment group, control group, methodological evaluation tools, outcome indicators, and principal conclusions.

### 2.4. Evaluation methods

#### 2.4.1. Report quality assessment tool—PRISMA statement

The PRISMA statement (22) consists of 27 entries in seven areas. Each entry is scored according to the degree of compliance according to the literature, with a score of 1 for “full report” if all of them are met, 0.5 for “partial report” if some are met, and 0 for “not reported” if none are mentioned. “The total score was 27.” When the literature score is 21–27, the report is considered relatively complete; when the score is 15–21, the message is deemed to be certain defects; when the score is 15 or less, the report is considered to have relatively severe information deficiency. Independent evaluators (WN-G and FY-X) independently appraised the report quality of the included SRs.

#### 2.4.2. Methodological quality assessment tool—AMSTAR2 scale

The AMSTAR2 (23, 24) scale contains 16 items, each of which is described by “yes” and “no” and some of which can be characterized by “partly yes.” Items 2, 4, 7, 9, 11, 13, and 15 are necessary. If there is no entry defect or only one non-critical entry defect, the methodological quality is high, and the system evaluation conclusion is accurate and comprehensive; if there is more than one non-critical entry defect but no critical entry defect, the methodological quality is medium, and the system evaluation conclusion is accurate; if there is one crucial entry defect with or without non-critical entry defect, the methodological quality is low, and the system evaluation conclusion If there is more than one critical entry defect with or without non-critical entry defects, the methodological quality is extremely low. The decision of this system evaluation is inaccurate and incomplete. Two authors (WN-G and FY-X) independently appraised the methodological quality of included SRs. The difference items after evaluation were discussed by a third evaluator and finally agreed upon.

#### 2.4.3. Evidence quality assessment—GRADE system

We assessed quality of evidence in included SRs using the GRADE system recommended in the Cochrane handbook (25) to comprehensively evaluate the quality level of the outcome indicators. We graded the quality of evidence for each main finding in each main comparison as “high” (no downgrade), “moderate” (downgraded by one level), “low” (downgraded by two levels), or “very low” (downgraded by three or more levels), based on judgments considering the following five factors: (1) limitation in study design and execution, (2) inconsistency of results, (3) indirectness of evidence, (4) imprecision, and (5) publication bias. Two authors (WN-G and FY-X) independently appraised the methodological quality of included SRs. The difference items after evaluation were discussed by a third evaluator and finally agreed upon. The GRADE was described in each outcome section.

### 2.5. Consistency test of assessment results

SPSS software (version 26.0) was used for data analyses. We used intraclass correlation coefficients (ICC) to test the consistency of the results of the two evaluators, including methodological quality and reportorial quality. We used a two-way random effects model with the average measurement to examine the ICC, in which a score of ≥0.75 was considered high in consistency, a score between 0.40 and 0.75 was considered to have moderate consistency, and a score of ≦0.4 was considered to have poor consistency. Categorical variables were reported as frequencies with percentages and descriptive analyses used to summarize the results. The statistical significance threshold was set at *p* < 0.05.

## 3. Results

### 3.1. Literature retrieval results

**Figure 1:**
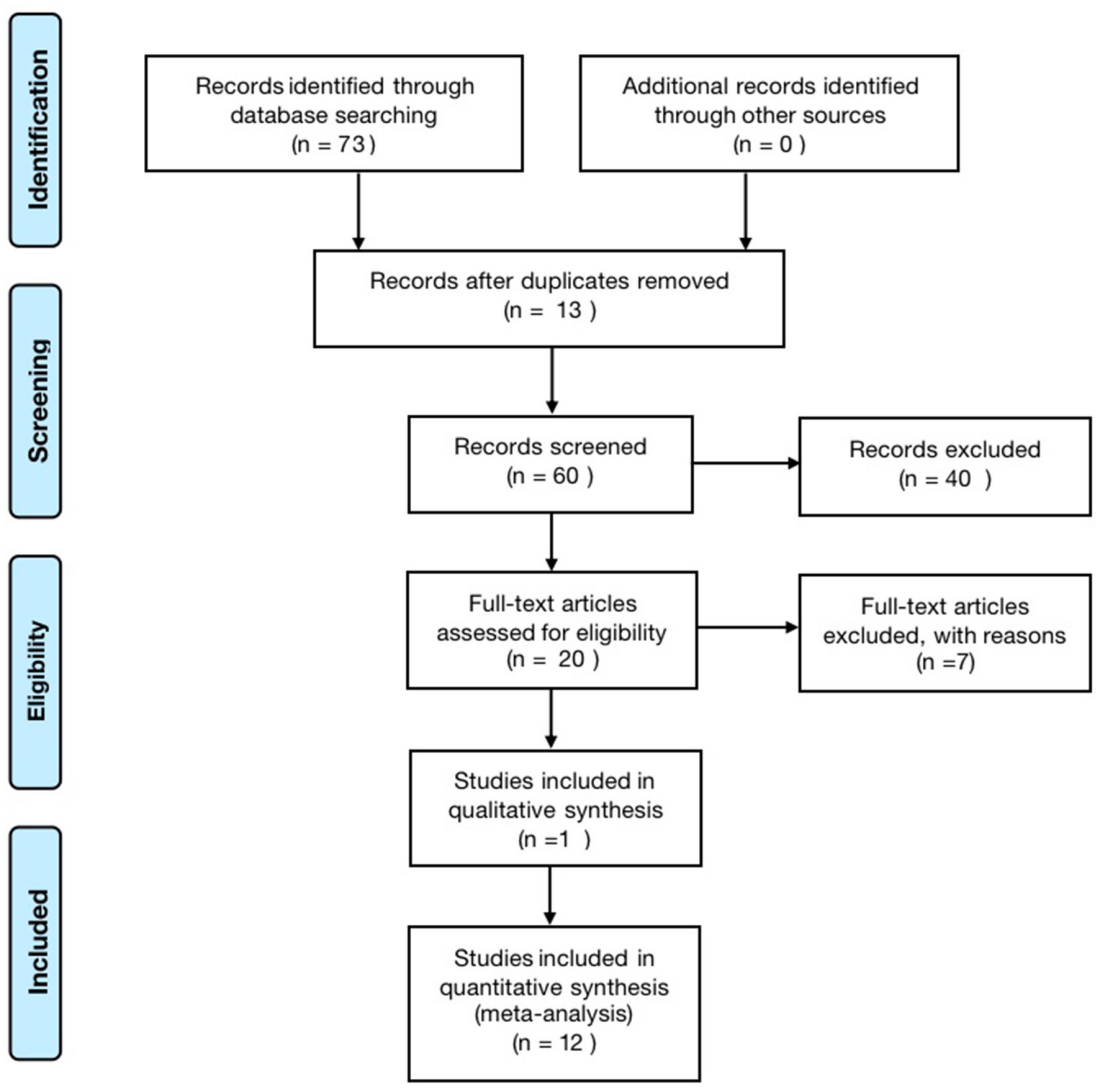
Flow chart of the literature search and study selection process.

According to the search strategy, 73 papers were retrieved, including 10 Meta-analyses and one qualitative analysis. The literature sources were six from China Knowledge Network, three from VIP, six from CBM, 13 from PubMed, 32 from the web of science, and 13 from Cochrane, excluding 13 duplicate publications, leaving 60. After reading the titles and abstracts, 40 literature with apparent discrepancies in literature type, study design, study subjects, and topics were excluded, and 20 were included; by reading the complete text, seven papers with inconsistent study designs, inconsistent topics or unclear descriptions were excluded, and 13 articles were finally included (26–38). The literature screening process is shown in Figure 1.

### 3.2. Basic features of the included literature

The essential characteristics of the included studies are shown in Table 1. Thirteen studies were included, seven in Chinese with literature distribution from 2015 to 2019 and six in English with literature distribution from 2011 to 2022. The most significant number of original studies included was 24, and the smallest was one; the largest sample size had was 2,562 cases, and the smallest was 92 cases; the interventions in the control group were mainly single or combination of antidepressants, sham stimulation, and conventional treatment, while the interventions in the experimental group were diversified, with unilateral high-frequency (>1 Hz) rTMS treatment, unilateral low-frequency (≤ 1 Hz) rTMS treatment, bilateral low-frequency (≤ 1 Hz) rTMS treatment, rTMS combined with antidepressants, rTMS combined with acupuncture, and other conventional therapies; the risk of bias evaluation tools were mainly Cochrane and PEDro scales; the outcome indicators were mainly efficiency, HAMD score, NIHSS score; in terms of conclusion, most of them believed that repetitive transcranial magnetic stimulation has some advantages in the treatment of post-stroke depression, but the results However, the results still need to be validated by more and higher quality studies.

**Table 1:** Characteristics of included systematic reviews.

**References**	**Country**	**Age**	**Study type**	**Number of RCTs/ number of cases**	**Therapy group**	**Control group**	**Basic features of repetitive transcranial magnetic**	**Methodology evaluation tools**	**Outcomes**	**Main conclusion**
Chen et al. (26)	China	No limit	Meta-analysis	16/1,208	rTMS, rTMS + routine treatment; rTMS+ ①/②/③/④/⑤/⑥/⑦/; rTMS+ acupuncture + routine treatment	Sham stimulation, sham stimulation + routine treatment, sham stimulation + ;①①/②/③/④/⑤/⑥/⑦/;+ routine treatment; routine treatment; routine treatment + rehabilitation treatment	3/5/10/20 HZ, left DLPFC;60–110%RMT; 10 days−8 weeks	Cochrane bias risk assessment	Recovery rate, HAMD, NIHSS, BI index, number of shedding cases, adverse events	High-frequency rTMS has good efficacy and acceptability in the treatment of PSD, but attention should be paid to adverse reactions such as headaches. Due to the limitations of the number and quality of the included studies, the above conclusions need to be verified by more high-quality studies
Lijun et al. (27)	China	No limit	Meta-analysis	13/1,033	rTMS	⑦/④/②/⑧/⑨/⑥	0.5/1/5/10 HZ, left DLPFC; 1 HZ, right DLPFC; 0.5 HZ bilateral prefrontal cortex; 60–100%RMT; 20–40 times	Cochrane Reviewer Handbook Quality evaluation criteria	HAMD, NIHSS, MMSE, Barthel index, modified Barthel index	Compared with antidepressants alone, rTMS combined with antidepressants may be more beneficial to improve the depressive symptoms of patients with post-stroke depression, as well as improve their neurological function, cognitive level, and daily living ability
Chaomeng et al. (28)	China	>20	Meta-analysis	18/1,376	rTMS + ④/⑥/②/⑩/①; rTMS; rTMS+ exercise therapy;routine treatment; rTMS+ ④ + psychological counseling	④/⑥/②/⑩/①;④ + psychotherapy; sham stimulation + exercise therapy; ② + routine treatment; routine treatment; routine treatment + psychological counseling	0.5/1/3/5/10 HZ, left DLPFC; 1 HZ, right DLPFC; 0.5/1 HZ bilateral prefrontal cortex; 60–110%RMT; 10 days−8 weeks	Cochrane bias risk assessment	HAMD, response rate, NIHSS, Barthel index	rTMS has a positive effect on the treatment of depression, neurological impairment, and impaired ability of daily living in PSD patients, and the above conclusions need to be further verified due to the quality level of included studies
Jin et al. (29)	China	>18	Meta-analysis	24/1,658	rTMS + ①/④/⑥/⑨/⑩; rTMS+ ①/④+ psychotherapy; rTMS, rTMS+ routine treatment of cerebrovascular diseases (withdrawal of antidepressants)	Sham stimulation + ① + psychotherapy; sham stimulation, sham stimulation + traditional therapy; conventional acupuncture; sham stimulation + antidepressant + physical therapy (music, etc.) + Traditional Chinese acupuncture + psychotherapy; ④/⑥/⑨/⑩	0.5/1/10/20 HZ, left DLPFC; 1 HZ, right DLPFC; 0.5/1 HZ bilateral prefrontal cortex; 60–110%RMT; 10 days−3 months	Modified Moher assessment scale	HAMD, response rate, NIHSS	rTMS has a positive effect on the mood of PSD patients. Due to the quality limitation of the included studies, the above conclusions still need to be verified by more large-sample, multi-center, and high-quality RCT studies
Chen et al. (30)	China	No limit	Meta-analysis	21/1,626	rTMS+ routine treatment, bilateral frontal rTMS+ ④/①/⑥ + routine treatment; rTMS+ acupuncture, rTMS+ ①/④/⑩/②/⑦+ routine treatment; rTMS+ acupuncture + Routine treatment; rTMS+ routine treatment; rTMS+ ④/⑦+ routine treatment	Sham stimulation + ① + routine treatment, routine treatment, sham stimulation + routine treatment, ①/④/⑨/②/⑦ + routine treatment	0.5/1 HZ, left DLPFC; 1 HZ, right DLPFC; 0.5/1 HZ bilateral prefrontal cortex; 60–100%RMT; 1–8 weeks	Cochrane bias risk assessment	HAMD, NIHSS, Barthel index, MMSE, adverse reactions	Low-frequency rTMS can significantly improve depression, daily living ability, and cognitive function of PSD patients, but there is insufficient evidence for NIHSS score improvement, and mild adverse reactions such as headache may occur, so high-quality clinical studies are needed to verify
Xiaojun and En (31)	China	No limit	Meta-analysis	3/81	rTMS	Antidepressants	Bilateral prefrontal cortex; left DLPFC; Bilateral occipital lobe	Cochrane bias risk assessment	Depressive symptoms	Repetitive transcranial magnetic stimulation can improve post-stroke depression to some extent. However, considering the lack of RCTs conforming to the criteria of a platoon and the imperfect methodological information, multi-center, and high-quality RCTs are needed to verify this conclusion in the future
Jing et al. (32)	China	No limit	Meta-analysis	9/664	rTMS	Blank control; sham stimulation	Low frequency rTMS, high frequency rTMS, low frequency rTMS+ high frequency rTMS, low frequency rTMS/high frequency rTMS are used alternately	Cochrane reviewer handbook quality evaluation criteria	HAMD, NIHSS, MESSS, adverse reactions, modified Barthel index	Repetitive transcranial magnetic stimulation (rTMS) can effectively improve the depressive state and quality of life before and after treatment, with small adverse reactions and no serious adverse reactions. However, the long-term effects of rTMS in the treatment of depression need further study
Shen et al. (33)	China	No limit	Systematic review, meta-analysis	22/1,764	rTMS + routine treatment; rTMS+ acupuncture + routine treatment; rTMS+ ④/⑦/⑨/⑥ + routine treatment; rTMS; rTMS+ acupuncture	Routine treatment, acupuncture + routine treatment, sham stimulation + routine treatment; antidepressants, sham stimulation;①/⑥/⑨; sham stimulation + antidepressants	Bilateral prefrontal cortex; motor cortex; left temporal parietal; high frequency rTMS, left DLPFC; Low frequency rTMSright DLPFC	Cochrane handbook for systematic reviews of interventions, grading of recommendations assessment, development, and evaluation	HAMD, response rate, remission rate, Barthel index, modified Barthel index, NIHSS Montgomery-asperger depression scale, depression questionnaire, safety	rTMS has a beneficial effect on PSD. However, due to heterogeneity and potential bias, these results should be treated with caution
Liu et al. (34)	China	No limit	Systematic review, meta-analysis	17/1,171	rTMS +/⑪/①/④/⑨/②/⑥/⑦+ routine treatment; rTMS+ routine treatment, rTMS	/⑪/①/④/⑨/②/⑥/⑦; antidepressants + routine treatment, sham stimulation + routine treatment, sham stimulation, routine treatment + rehabilitation treatment	10 HZ, left DLPFC; 60–110%RMT; 2–12 weeks	Physiotherapy evidence database scoring system	HAMD, response rate, remission rate, Barthel index, NIHSS, adverse reactions, and number of dropouts	rTMS is an effective intervention for post-stroke depression, but the safety of treatment needs to be further verified by a large sample multicenter trial
Shao et al. (35)	China	No limit	Systematic review, meta-analysis	7/351	rTMS+ routine treatment, rTMS+ ④ + routine treatment, rTMS, rTMS+ ①	④+ routine treatment, routine treatment, sham stimulation, sham stimulation +①	Bilateral-rTMS; Unilateral-rTMS; left DLPFC 10 Hz; right DLPFC 1 Hz; 1–4 weeks	Cochrane handbook for systematic reviews of interventions	HAMD, NIHSS, remission rate, MMSE	rTMS may be an effective treatment for PSD patients. Further clinical studies with larger sample sizes and clearer subgroup definitions are needed to confirm these results
McIntyre et al. (36)	Canada	No limit	Systematic review	1/92	rTMS	Blank control, sham stimulation	1/10 HZ, left DLPFC; 100–110%RMT; 10 sessions	Physiotherapy evidence database scoring system	HAMD, response rate, the remission rate	Transcranial magnetic stimulation is beneficial in the short term for the treatment of depression in patients after stroke. However, heterogeneity of population, variability of study design and protocol, and limited number of studies affect the reliability of this conclusion
Shen et al. (37)	China	No limit	Systematic review, meta-analysis	7/258	rTMS	Sham stimulation	10 HZ, left DLPFC; 1 HZ leftDLPFC + 10 HZ right DLPFC; 80–110%RMT	Cochrane risk of bias tool	NIHSS, Modified rankin scale	rTMS and tDCS were demonstrated to be effective and safe treatment techniques for PSD. More large-scale studies were essential to explore the effect of rTMS with different frequencies and tDCS on PSD.
Liang et al. (38)	China	No limit	Systematic review, meta-analysis	32/2,562	rTMS +④/②/⑤/⑨/①/⑪/	④/②/⑤/⑨/①/⑪/	1/3/5/10/20 HZ, left DLPFC; 1 HZ right DLPFC; 3 HZ left temporo-parietal lobe; 21 HZ parietal CZ region posterior 1 cm; 1 HZ bilateral dorsolateral forehead; 0.5 HZ bilateral prefrontal lobes; 80–120%RMT; 7–6 days	Cochrane collaboration's tool	HAMD, total effective rate, MBI score, adverse effects	Low-frequency rTMS combined with antidepressants tends to be more effective than antidepressants alone in patients with PSD, and there are no significant adverse effects. In addition, combined therapy may enhance quality of life after stroke. Combination therapy with high-frequency rTMS (>10 Hz) showed no advantage in treating PSD. The transcranial electrical stimulation (TES) combined with antidepressants might be more effective than antidepressants alone

Antidepressant: ① lupentixton merritol; ② escitalopram; ③ Chaihushugan SAN; ④ fluoxetine; ⑤ venlafaxine; ⑥ duloxetine; ⑦ sertraline; ⑧ citalopram hydrobromide; ⑨ mirtazapine; ⑩ zoloft; ⑪ paroxetine.

### 3.3. Report the quality evaluation results

The PRISMA statement score of the included literature ranged from 12 to 22.5, including three reports with relative completeness, eight reports with certain defects, and two reports with severe information deficiency. PRISMA declared item reporting, 13 pieces of literature were fully reported in the title (Item 1) and partially reported in the structured abstract (Item 2). In the preface, 13 articles elaborate on the theoretical basis (item 3), and six pieces complete the report's purpose (Item 4). In the part of methods, protocol, and registration (Item 5), there are two complete reports, 13 complete reports on information sources (item 7) and research selection process (Item 9), and partial reports on inclusion criteria (Item 6) and data items (Item 11). Single study bias (Item 12), summary effect indicators (Item 13), and plan analysis methods (Item 14) were reported in total for most of them. Other analyses (Item 16) were reported for 10 pieces of literature. In the results section, complete reports were made on research characteristics (Item 18) of all 13 works of literature, as well as research selection (Item 17), risk of internal bias (Item 19), single research results (Item 20), and integration of results (Item 21) of most pieces of literature. In the discussion section, all literature partially reports on the summary of evidence (item24) and 13 literature fully report on the limitations (item 25). For funding sources (item 27) only a few literatures partially report. See Table 2 for details.

**Table 2:** PRISMA declaration entries report conditions.

	**Item 1**	**Item 2**	**Item 3**	**Item 4**	**Item 5**	**Item 6**	**Item 7**	**Item 8**	**Item 9**	**Item 10**	**Item 11**	**Item 12**	**Item 13**	**Item 14**
Y	13	0	13	6	2	0	13	6	13	2	0	11	12	12
PY	0	13	0	7	0	13	0	0	0	3	13	0	0	0
N	0	0	0	0	11	0	0	7	0	8	0	2	1	1
	**Item 15**	**Item 16**	**Item 17**	**Item 18**	**Item 19**	**Item 20**	**Item 21**	**Item 22**	**Item 23**	**Item 24**	**Item 25**	**Item 26**	**Item 27**	
Y	0	10	11	13	11	12	12	3	8	0	11	13	0	
PY	0	0	1	0	0	0	0	1	1	13	0	0	4	
N	13	3	1	0	2	1	1	9	4	0	2	0	9	

Item 1 title; item 2 structured abstract; item 3 theoretical basis; item 4 introductory purpose; item 5 protocol and registration; item 6 inclusion criteria; item 7 sources of information; item 8 search; item 9 study selection; item 10 data extraction; item 11 data item; item 12 individual study bias; item 13 effect indicators; item 14 synthetic outcome methods; item 15 inter-study bias methods item 16 supplemental analysis methods; item 17 study screening; item 18 study characteristics; item 19 risk of intra-study bias; item 20 individual study results; item 21 synthetic results; item 22 inter-study bias results; item 23 supplemental analysis results; item 24 evidence summary; item 25 limitations; item 26 conclusions; item 27 funding.

### 3.4. Evaluation results of methodological quality

AMSTAR2 scale was used to evaluate the methodology of the 13 included pieces of literature. The results showed that at least two critical items in each literature failed to reach the standard, and the methodological quality was deficient. Among the essential items, the compliance rate of Item 2 was 15.3%, nine articles partially met the requirements of Item 4, 0% of Item 7, 92% of item 9, all pieces in Item 11 reached the standard, Item 13 compliance rate of 77%, Item 15 compliance rate of 7.7%. Among the non-key items, Item 1 all meet the standard, 83.33% of Item 6 reached the middle, 0% of Item 3 and 16 got the standard, 92% of Item 5 came the standard, Item 8 had 12 documents partially met the standard, Item 12 met the standard with six articles and did not meet the standard with seven articles, and Item 14 met the standard with 61.5%. For details, see Table 3.

**Table 3:** AMSTAR2 scale evaluation results.

**References**	**Item 1**	Item 2*	**Item 3**	Item 4*	**Item 5**	**Item 6**	Item 7*	**Item 8**	Item 9*	**Item 10**	Item 11*	**Item 12**	Item 13*	**Item 14**	Item 15*	**Item 16**
Chen et al. (26)	Y	N	N	PY	Y	Y	N	PY	Y	N	Y	N	Y	N	N	N
Li et al. (27)	Y	N	N	PY	N	Y	N	PY	Y	PY	Y	N	N	N	N	N
Liu et al. (28)	Y	N	N	PY	Y	Y	N	Y	Y	PY	Y	N	Y	Y	N	N
Jin et al. (29)	Y	N	N	PY	Y	N	N	PY	Y	PY	Y	N	Y	Y	N	N
Chen et al. (30)	Y	N	N	PY	Y	Y	N	PY	Y	N	Y	Y	Y	Y	N	N
Ding and En (31)	Y	N	N	PY	Y	Y	N	PY	Y	N	Y	N	Y	Y	N	N
Zhang et al. (32)	Y	N	N	PY	Y	Y	N	PY	Y	N	Y	N	N	N	N	N
Shen et al. (33)	Y	Y	N	N	Y	Y	N	PY	Y	PY	Y	Y	Y	Y	N	N
Liu et al. (34)	Y	Y	N	N	Y	Y	N	PY	N	PY	Y	Y	Y	Y	N	N
Shao et al. (35)	Y	N	N	N	Y	Y	N	PY	Y	PY	Y	Y	N	N	N	N
McIntyre et al. (36)	Y	N	N	N	Y	N	N	PY	Y	N	Y	N	N	N	N	N
Shen et al. (37)	Y	N	N	PY	Y	Y	N	PY	Y	Y	Y	Y	Y	Y	Y	N
Liang et al. (38)	Y	N	N	PY	Y	Y	N	PY	Y	N	Y	Y	Y	Y	N	N

^*^Y, yes; N, no; PY, partial yes; item 1: Do the study questions and inclusion criteria include PICO? item 2: Is there a pre-published protocol? Is there significant bias between the study and the protocol? item 3: Did the authors explain the type of study design included? item 4: Was a comprehensive literature search strategy used? item 5: Was duplicate study screening performed? item 6: Were duplicate data extractions performed? item 7: Was a list of excluded literature provided, with reasons for the exclusion? item 8: Was a detailed description of the included studies provided? item 9: Was the risk of bias for each included study assessed using a reasonable tool? item 10: Is the source of funds for the included studies reported? item 11: If Meta-analyses were performed, were the results statistically combined using appropriate methods? item 12: If Meta-analyses were performed, is the effect of risk of bias described in the results? item 13: If a Meta-analysis was performed, is the effect of risk of bias described in the discussion? item 14: Is heterogeneity justified in the discussion? item 15: If a quantitative analysis was performed, was publication bias adequately investigated and its possible impact discussed? item 16: Are any potential sources of conflict of interest reported?

### 3.5. Evidence quality level evaluation results

The GRADE system was used to rate the quality of evidence, and 13 papers contained 42 pieces of evidence for outcome indicators. The results showed no high-level evidence in the included literature, 8 (19%) medium-level evidence, 12 (28.5%) low-level evidence, and 22 (52.4%) very-low-level evidence, which was generally of low quality. The reasons are as follows: (1) Serious flaws in the blinded implementation, allocation concealment, and randomization procedures of the randomized controlled trials contained in the included literature compromise the validity of the findings. (2) The reliability of the results is impacted by the heterogeneity of several of the included outcome indicators. (3) Fewer included studies, smaller sample sizes, and incomplete consistency in selecting interventions, data extraction, and outcome indicators all affect The accuracy of the results. (4) The possibility of publication bias. For details, see Table 4.

**Table 4:** Quality of evidence in included systematic reviews with GRADE.

**References**	**Outcomes (number of studies)**	**Publication bias**	**Risk of bias**	**Imprecision**	**Inconsistency**	**Indirectness**	**Quality of evidence**
Chen et al. (26)	HAMD (24)	−1^a^	−1^b^	0	0	0	Low
	BI (8)	−1^a^	−1^b^	0	0	0	Low
	NIHSS (5)	−1^a^	−1^b^	0	0	0	Low
	ER (9)	−1^a^	−1^b^	0	0	0	Low
Li et al. (27)	BI (6)	−1^a^	−1^b^	0	0	0	Low
	NIHSS (3)	−1^a^	−1^b^	−1^c^	0	0	Very low
	HAMD (13)	−1^a^	−1^b^	0	0	0	Low
	MMSE (2)	−1^a^	−1^b^	−1^c^	0	0	Very low
Liu et al. (28)	HAMD (17)	0	−1^b^	0	−2^e^	0	Very low
	NIHSS (4)	0	−1^b^	−1^c^	0	0	Low
	ER (5)	0	−1^b^	−1^c^	0	0	Low
	BI (7)	0	−1^b^	0	−2^e^	0	Very low
Jin et al. (29)	HAMD (24)	0	−1^b^	0	−2^e^	0	Very low
	NIHSS (2)	0	−1^b^	0	0	0	Moderate
	ER (11)	0	−1^b^	0	0	0	Moderate
Chen et al. (30)	HAMD (21)	−1^a^	−1^b^	0	−1^d^	0	Moderate
	BI (11)	−1^a^	−1^b^	0	0	0	Low
	NIHSS (4)	−1^a^	−1^b^	−1^c^	0	0	Very low
	MMSE (6)	−1^a^	−1^b^	0	0	0	Low
Ding and En (31)	DS (3)	−1^a^	−1^b^	−1^c^	−1^d^	0	Very low
Zhang et al. (32)	HAMD (9)	−1^a^	−1^b^	0	−2^e^	0	Very low
	NIHSS (3)	−1^a^	−1^b^	−1^c^	0	0	Very low
	MBI (5)	−1^a^	−1^b^	−1^c^	0	0	Very low
	MESSS (1)	−1^a^	−1^b^	−1^c^	0	0	Very low
Shen et al. (33)	HAMD (24)	0	−1^b^	0	0	0	Moderate
	ER (12)	−1^a^	0	0	0	0	Moderate
	BI (7)	0	−1^b^	−1^c^	0	0	Low
	MARDS (1)	0	−1^b^	0	0	0	Moderate
Liu et al. (34)	HAMD (15)	0	−1^b^	0	−2^e^	0	Very low
	BI (3)	0	−1^b^	−1^c^	−2^e^	0	Very low
	NIHSS(4)	0	−1^b^	−1^c^	0	0	Low
	ER (8)	0	−1^b^	0	0	0	Moderate
Shao et al. (35)	HAMD (7)	−1^a^	−1^b^	0	−1^d^	0	Very low
	NIHSS (1)	−1^a^	−1^b^	−1^c^	−1^d^	0	Very low
	MMSE (2)	−1^a^	−1^b^	−1^c^	−1^d^	0	Very low
McIntyre et al. (36)	HAMD (5)	−1^a^	−1^b^	−1^c^	0	0	Very low
	ER (5)	−1^a^	−1^b^	−1^c^	0	0	Very low
Shen et al. (37)	NIHSS (3)	0	−1^b^	−1^c^	−2^e^	0	Very low
	MRS (3)	0	−1^b^	−1^c^	−2^e^	0	Very low
Liang et al. (38)	HAMD (34)	0	−1^b^	0	−2^e^	0	Very low
	ER (17)	0	−1^b^	0	0	0	Moderate
	MBI (7)	0	−1^b^	0	−2^e^	0	Very low

BI, Barthel index; MBI, modified Barthel index; ER, effective rate; MADS, Montgomery-Asperger Depression Scale; BECK, BECK Self-rating Depression Questionnaire; DS, Depressive symptoms; MESSS, modified Scandinavian Stroke Scale; MRS, Modified Rankin Scale.

a Asymmetric funnel plots or all positive results may have a large publication bias.

b Risk of bias in included studies with respect to randomization, blinding, allocation concealment, completeness of outcome data, or selective reporting risk of bias.

c Inclusion of too small a sample size for the study (sample size for continuous variables < 400).

d Included in the study 50% ≤ I^2^ < 75%.

e Included in the study 75% ≤ I^2^ < 100%.

### 3.6. Consistency test of assessment results

**Figure 2:**
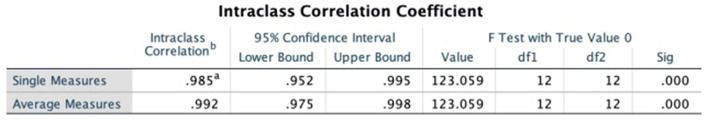
Intraclass correlation coefficient of PRISMA declaration. ^*a, b*^Represent the two persons who performed the cross-evaluation, WG and FX.

**Figure 3:**
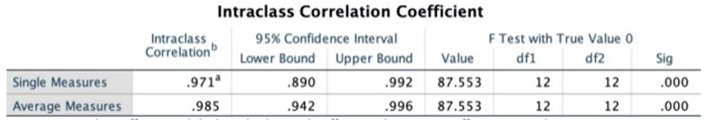
Intraclass correlation coefficient of AMSTAR2 scale. ^*a, b*^Represent the two persons who performed the cross-evaluation, WG and FX.

**Figure 4:**
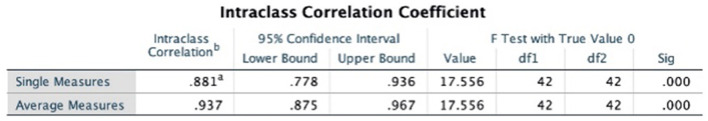
Intraclass correlation coefficient of GRADE system. ^*a, b*^Represent the two persons who performed the cross-evaluation, WG and FX.

The intraclass correlation coefficients (ICC) was used to test the consistency of the results evaluated by the two evaluators, and the results of both evaluators for methodological quality, report quality, and evidence quality were highly consistent, as detailed in Figures 2–4.

### 3.7. Main outcome measures

Most current studies have focused on the efficacy and safety of repetitive transcranial magnetic stimulation in patients with post-stroke depression in terms of improvement in the following areas: effective clinical efficacy, depressive symptoms, neurological function, cognitive function, activities of daily living, and adverse effects.

#### 3.7.1. Effective clinical efficacy

Of the 13 included papers, four analyzed the clinical efficacy of repetitive transcranial magnetic stimulation for post-stroke depression. With a total of 3,457 patients and 54 RCTs enrolled, interventions were divided into six types: (1) repetitive transcranial magnetic stimulation alone (2) repetitive transcranial magnetic stimulation + conventional therapy (3) repetitive transcranial magnetic stimulation + antidepressant medication + conventional therapy (4) repetitive transcranial magnetic stimulation + acupuncture (5) repetitive Transcranial magnetic stimulation + antidepressants (6) repetitive transcranial magnetic stimulation + antidepressants + psychotherapy; control measures were divided into six, four studies were positive controls, and 38 studies were inert controls. All studies reported higher efficiency of rTMS as an intervention for psd than the control group.

#### 3.7.2. Improvement of depressive symptoms

Of the 13 included papers, 184 RCTs had HAMD score as an outcome indicator, with a total of 13,505; the interventions were divided into eight types: (1) repetitive transcranial magnetic stimulation treatment alone, (2) repetitive transcranial magnetic stimulation + conventional treatment, (3) repetitive transcranial magnetic stimulation + antidepressant medication + conventional treatment, (4) repetitive transcranial magnetic stimulation + acupuncture treatment, (5) repetitive transcranial magnetic stimulation + antidepressants, (6) repetitive transcranial magnetic stimulation + antidepressants + psychotherapy, (7) repetitive transcranial magnetic stimulation + acupuncture + conventional treatment, (8) repetitive transcranial magnetic stimulation + herbal treatment + conventional treatment; control measures were divided into two types, 15 studies were positive controls and 135 studies were inert controls. All studies reported higher HAMD scores in the treated group than in the control group after treatment.

#### 3.7.3. Recovery of neurological function

Of the 13 included papers, 56 RCTs had NIHSS scores as an outcome indicator, with a total of 2,850; interventions were divided into six types: (1) repetitive transcranial magnetic stimulation treatment alone, (2) repetitive transcranial magnetic stimulation + antidepressant medication + conventional treatment, (3) repetitive transcranial magnetic stimulation + antidepressant medication, (4) repetitive transcranial magnetic stimulation + conventional treatment, (5) repetitive transcranial magnetic stimulation + antidepressants + psychotherapy, (6) repetitive transcranial magnetic stimulation + acupuncture therapy + conventional therapy; control measures were divided into six, 30 studies were inert controls. All studies showed that the treatment group was more effective in reducing patients' NIHSS scores than the control group.

#### 3.7.4. Improvement of cognitive function

Three of the included articles addressed the effects of repetitive transcranial magnetic stimulation on cognitive function in depressed patients after stroke, all assessed by the MMSE scale. With a total of 778 patients and 10 RCTs enrolled, interventions were divided into two types: (1) repetitive transcranial magnetic stimulation + antidepressant medication + conventional therapy, (2) repetitive Transcranial magnetic stimulation + antidepressants. Two of these studies showed that the treatment group significantly improved cognitive function in post-stroke depression patients compared to the control group. One of the studies reported no significant difference in the recovery of cognitive function between patients in the treatment and control groups after treatment.

#### 3.7.5. Improvement of the ability to perform activities of daily living

Six of the included articles addressed the effect of repetitive transcranial magnetic stimulation on the ability to perform activities of daily living in depressed patients after stroke, as assessed by the BI or MBI scales. With a total of 3,112 patients and 6 RCTs enrolled, interventions were divided into five types: (1) repetitive transcranial magnetic stimulation treatment alone, (2) repetitive transcranial magnetic stimulation + antidepressant medication + conventional treatment, (3) repetitive transcranial magnetic stimulation + antidepressant medication, (4) repetitive transcranial magnetic stimulation + conventional treatment, (5) repetitive transcranial magnetic stimulation + antidepressants + psychotherapy. All four studies showed that the treatment group improved the patient's daily living activities more effectively than the control group. However, two studies reported no significant difference between the treatment and control groups regarding the recovery of patients' activities of daily living after treatment.

#### 3.7.6. Adverse effects

Eight of the included articles addressed adverse effects after repetitive transcranial magnetic stimulation therapy. With a total of 4,822 patients and 8 RCTs enrolled, interventions were divided into six types: (1) repetitive transcranial magnetic stimulation treatment alone, (2) repetitive transcranial magnetic stimulation + antidepressant medication + conventional treatment, (3) repetitive transcranial magnetic stimulation + antidepressant medication, (4) repetitive transcranial magnetic stimulation + conventional treatment, (5) repetitive transcranial magnetic stimulation + antidepressants + psychotherapy, (6) repetitive transcranial magnetic stimulation + acupuncture therapy + conventional therapy. Six studies showed that the treatment group had a higher probability of adverse reactions after treatment, and the highest proportion of adverse reactions was a headache. Two studies reported that the treatment and control groups had no significant difference in adverse reactions after treatment. Other recorded adverse effects, such as loss of appetite, local discomfort, anxiety, fatigue, and dry mouth, were not significantly different between the treatment and control groups.

## 4. Discussion

Multiple post-stroke functional deficits, depressed symptoms, and somatic signs of depression are all present in patients with post-stroke depression. On the one hand, stroke-related motor and cognitive dysfunction affects the execution of treatment plans for depression and lowers their effectiveness; on the other hand, depression lowers the motivation and initiative of stroke patients in rehabilitation, which affects the efficacy of rehabilitation treatment and delays the recovery process. The functional damage brought on by depression and stroke combined is more severe, and the effects on families and society are far more detrimental. The success of conventional post-stroke depression treatments no longer matches the patients' health needs as a result of the bio-medical-psychological paradigm change, necessitating the urgent search for safer, more efficient, and more acceptable treatments.

The mechanism of repetitive transcranial magnetic stimulation for post-stroke depression is not well-understood clinically, although the more widely used therapies include: (1) Enhancing the brain environment, decreasing the inflammatory response, and protecting the nerves: rTMS can improve the state of reduced glucose metabolism, improve the hemodynamics of ischemic brain tissue and increase collateral circulation, reduce oxidative stress of the neuroimmune system and inhibit the production of inflammatory factors, and produce neuroprotective effects by controlling the levels of redox mediators. (2) Modulation of the brain's chemical alterations associated with depression: rTMS can have intricate neurochemical effects that control neurotransmitter release and modulate the expression of receptors that are involved. The γ-aminobutyric acid system can be activated, monoamine neurotransmitters can be elevated, and subventricular zone proliferation of neural stem cells can be induced by rTMS (39). The study discovered that serum levels of brain-derived neurotrophic factor were significantly lower in PSD patients than in patients without depression, and that rTMS, antidepressants, and aerobic exercise could increase the brain's expression of Brain-derived neurotrophic factor to lessen depressive symptoms (40). rTMS could also encourage the release of more dopamine in the striatum and hippocampus of PSD patients. hippocampus to release more dopamine, norepinephrine, 5-hydroxytryptamine, and other neurotransmitters, lowering the number of norepinephrinergic receptors per unit of cerebral cortex while modulating neurotransmitter content, and weakening the sensitivity of 5-hydroxytryptamine receptors in the post-synaptic membrane of the hypothalamus, relieving depression (33). (3) Cortical network rebuilding intervention: It was discovered that rTMS has more distinct antidepressant mechanisms in addition to similar effects to those of antidepressant drugs (correction of catecholamine and its receptors, improvement of brain nutrition and development, and reduction of inflammatory factors in the brain). For instance, rTMS not only stimulates the left prefrontal cortex, anterior cingulate gyrus, basal ganglia, and hippocampus but also increases the volume of the stimulated lateral hippocampus and the thickness of the anterior cingulate cortex, thereby regulating cortical excitability, enhancing cerebral blood flow and metabolism, and restoring cortical network function. (4) Influence on neuroplasticity: The restoration of neurological function after a stroke is correlated with the rupture of the mutual inhibition balance mechanism that predominates between the hemispheres. Increased local cerebral blood flow and velocity, the expression of functional synaptic plasticity, and the reconstruction of cortical function to create new conduction pathways are all effects of rTMS stimulation of the cerebral cortex. Both low-frequency rTMS stimulation of the healthy hemisphere to reduce the relatively dominant inhibitory function and high-frequency rTMS stimulation of the damaged hemisphere to reactivate areas lacking excitatory activity can result in “from the establishment of newly connected individual neuronal pathways to systemic adjustments” that affect neuroplasticity and improve the loss of interhemispheric inhibition. The imbalance of mutual inhibition between the hemispheres is improved, thus facilitating the recovery of neurological function after stroke.

Our study discovered that rTMS also enhanced the cognitive function, and daily living skills of PSD patients, indicating that rTMS can boost patient initiative and encourage active rehabilitation exercises, which is noteworthy for enhancing the general effectiveness of stroke patients' rehabilitation. Additionally, a number of RCTs used the NIHSS score, Modified Rankin Scale, and total effectiveness to assess the effectiveness of the treatment for PSD patients. The findings indicate that rTMS not only reduced depressive symptoms but also somewhat reduced neurological deficits and overall functional condition in stroke patients, In other words, independent of the rTMS-related therapies performed, the global efficacy of the treatment of PSD patients improved, this is an exciting conclusion.

The current study summarized the characteristics of the present clinical application of repetitive transcranial magnetic stimulation for post-stroke depression: (1) Treatment frequency: 1 Hz is mainly chosen for low-frequency treatment and 10Hz for high-frequency treatment. (2) Treatment site: Almost all clinical studies chose the treatment site as the dorsolateral prefrontal lobe, but the frequency of treatment and the treatment site is not fixed together. (3) Intensity: The treatment intensity of 60–110% RMT is currently the most applied intensity selected for clinical treatment. (4) The duration of treatment spans a wide range, with the shortest being 7 days and the longest being 3 months. (5) In our investigation, it was discovered that a significant portion of current RCT treatment protocols contained thorough explanations of the “frequency” and “treatment site” components, the details on the quantity of rTMS treatments, the quantity of pulses, the length of each treatment, and the overall treatment period were rather thorough. However, the details of the treatment protocol for repetitive transcranial magnetic stimulation are poorly described, such as the data on the number of pulses per train, the number of trains, and the time between each train.

The present study also noted a few restrictions on the TMS for PSD clinical trials currently being conducted today: (1) Since most RCTs used drug-controlled comparisons across groups and treated patients with both rTMS and drugs rather than just a single rTMS session, it is still unclear if the findings from this experimental strategy can conclusively show that rTMS has a therapeutic effect on PSD. (2) The majority of studies did not specify whether there was enough of a run-in period to rid patients of any other medications they might have taken, to allow the body to clear prior treatments that might have affected the study, and to rule out the possibility that medications taken before the clinical study might have interacted with the study medications to cause toxic side effects, while also ensuring that the patients' true conditions were being taken into account. (3) Transcranial magnetic stimulation is a sophisticated non-pharmacological intervention that necessitates a thorough explanation of a lot of different parts in order to make the precise treatment plan clear (41–45). (4) The comfort modality for the control group in the ongoing placebo control study of recurrent transcranial magnetic stimulation is sham stimulation. Nevertheless, it is more challenging for both the operator and the patient to be in a blinded state during the therapy due to the specific properties of TMS operation. For instance, reversing the stimulation coil to provide the sham stimulation used in the majority of treatments increases the possibility that the operator or the patient will be aware of the treatment. It is also known that TMS therapy may cause movements of the jaw joints or facial muscles. Experienced operators can also determine if the patient is receiving real or sham stimulation using these displays. Future non-invasive brain stimulation clinical trials should tighten the requirement for “sham stimulation” in practice, according to a 2022 study on non-invasive brain stimulation and neuroenhancement. As with any technique, the accuracy of the experimental results depends on the equipment and how well this is matched to the experience and skill of the operator (46). (5) Psychotherapy is another potential intervention that can be considered for treating depression in conjunction with pharmacotherapy. Unfortunately, there aren't enough randomized, controlled studies demonstrating the efficacy of PSD psychotherapy. Only three trials were included in a meta-analysis in 2008 (5), and no pooled advantage was found. The American Heart Association concluded that brief psychosocial therapies might be beneficial but need empirical confirmation based on an overall assessment of these and more current trials (47).

Additionally, the highest proportion of adverse reactions to treatment recorded in the literature included in the current study was headache, and patients are able to finish their treatment after rest or symptomatic relief. Although the safety of rTMS therapy is assured, should high-frequency rTMS be avoided at the location of the lesion and used cautiously given the elevated risk of seizures following cortical stroke? The only absolute contraindications to rTMS mentioned in the 2009 version and 2021 update of the guidelines (48, 49) for the use of TMS is the presence of metallic hardware in close contact to the discharging coil (such as cochlear implants, or an Internal Pulse Generator or medication pumps). Vascular lesions are another potential epilepsy risk factor, but no specific time period following a stroke was specified as prohibited. There have only been a few studies using transcranial magnetic stimulation in patients with acute stroke because the clinical use of repetitive transcranial magnetic stimulation for the treatment of post-stroke dysfunction is still constrained by the time of onset of patients in the inclusion criteria. On the negative effects and safety of transcranial magnetic stimulation in patients with acute stroke, there is still no clinical agreement. There are no guidelines to provide treatment protocols for patients with PSD, whose conventional treatment for stroke conditions is complex and uninterruptible, and which must be taken into account when designing treatment plans. In the RCTs included in this study, rTMS is primarily used as an adjunctive measure, and the determination of stimulation parameters such as frequency, site, and duration of stimulation is mostly empirical and does not take into account individual differences. We recommend that future clinical research consider the unique characteristics of PSD patients as well as the results of this investigation.

The findings of this study, which assessed the systematic evaluations of the included studies using the PRISMA statement, the AMSTAR2 scale, and the GRADE system, show limitations that are present in currently published systematic evaluations/meta-analyses of repetitive transcranial magnetic stimulation for post-stroke depression: only two SRs provided pre-protocols, and nearly all SRs did not search for relevant gray literature and trial registry websites at the time of the search. The methodological quality of all included SRs was extremely poor due to the lack of a list of rejected literature, potential omissions in research screening, and inadequate analysis of publication bias. The majority of SRs did not provide the funding source for the included studies, and most did not provide a description of potential confounding factors. In addition, almost all SRs did not explain the type of included studies in a manner consistent with their inclusion, even though the included ones were RCTs. This had some impact on the rigor of SRs as the strongest source of evidence. These issues have an impact on both the utilization of rTMS in clinical settings as well as the validity of the systematic evaluation itself. Numerous elements that increase bias and heterogeneity were incorporated into the majority of the studies' designs, including blinding procedures, allocation concealment, and randomization techniques. The dearth of recent high-quality RCT trials on rTMS for PSD is also directly attributed to the inclusion of fewer studies, lower sample sizes, and inconsistent choice of therapies, data extraction, and outcome markers. Heterogeneity may also be influenced by the variety of rTMS therapy parameters and their combination with other therapies, as well as patient individuality, psychological health, stroke kind, and stroke severity. The principal causes of this include This study suggests that in order to address the aforementioned problems, future researchers should attempt to avoid the aforementioned shortcomings when conducting systematic evaluation studies of depression following repeated transcranial magnetic stimulation for stroke, as well as standardizing the design and study of experiments, developing pre-protocols, and registering them; enact a thorough search approach, concentrating on the gray literature and languages other than English and Chinese; and, when appropriate, screen original studies strict gatekeeping to enhance the standard of original research included in the systematic evaluation and offer a list of excluded literature and justifications for exclusion; elaborate on the key features of the included literature to make it easier to find sources of heterogeneity; Analyze the influence of the risk of bias on the study results and explain and debate the impact of publication bias. Results from systematic evaluations with less bias are more trustworthy.

This overview has some limitations. Although investigator cross-evaluation was repeated throughout the process, it also produced subjective results; the interventions included in the study were complex, and their effect values could not be quantitatively combined and analyzed; etc. The results of this study were obtained from the subjective evaluation of the investigators, and only qualitative analysis was conducted; quantitative evaluation was not possible. Therefore, we propose that while using this data for clinical decision making, clinical personnel should make reference to the real scenario.

## 5. Conclusion

Repetitive transcranial magnetic stimulation for post-stroke depression was effective in improving patients' depressive symptoms and neurological function, according to the systematic review included in the current study. However, only one of the included original studies (50) underwent long-term follow-up, so the potential for long-term effects of rTMS for PSD still needs to be further explored. To propose a standardized management protocol for repetitive transcranial magnetic stimulation and to investigate how rTMS can naturally be integrated with other rehabilitation therapies for patients with PSD, follow-up high-quality, large-scale, and multicenter studies on the application of rTMS for PSD are still required.

In summary, the present study concludes by summarizing the systematic analyses of depression after stroke that have been treated with repetitive transcranial magnetic stimulation. The quality of reporting and evidence, as well as the methodology used in the current systematic evaluations, can all be much improved. Due to the small number of included articles, methodological issues, potential bias risks, and significant heterogeneity, which lower the reliability of the results, please proceed with caution when interpreting the conclusions of this study.

## Data availability statement

The original contributions presented in the study are included in the article/Supplementary material, further inquiries can be directed to the corresponding authors.

## Author contributions

WG: conceptualization, methodology, formal analysis, writing—original draft, and supervision. WZ: conceptualization, data curation, supervision, validation, and writing—review and editing. FX: conceptualization, methodology, formal analysis, and supervision. BY and SY: data curation, formal analysis, and methodology. HH: funding acquisition and writing—review and editing. All authors contributed to the article and approved the submitted version.
