# Supplementary material for: Repetitive transcranial magnetic stimulation for post-stroke depression: An overview of systematic reviews
Source: Front Neurol. 2023 Mar 16;14:930558. doi: 10.3389/fneur.2023.930558 (PMC10061017; doi:10.3389/fneur.2023.930558)
Supplement: Supplementary file 1 [file Table_1.docx]

**Appendix a**

#1 "Repetitive transcranial magnetic stimulation" [Mesh]
#2 "Transcranial magnetic stimulation" [Mesh]

#3 noninvasive brain stimulation

#4 #1 OR #2 OR #3

#5" Depression "[Mesh]

#6" Depressed "[Mesh]

#7" Post-stroke depression "[Mesh]

#8 #5 OR #6 OR #7

#9" stroke"[Mesh]

#10 brain vascular accident

#11 #9 OR #10 OR
#12" meta analysis "[Mesh]

#13" systematic review "[Mesh]
#14" systematic assessment "[Mesh]

#15" system evaluation "[Mesh]

#16" systematic evaluation "[Mesh]

#17"systematic review "[Mesh]

#18"systematical review"[Mesh]

#19 #11 OR #12 OR #13 OR #14 OR #15 OR #16 OR #17 OR #18
#20 #4 AND #8 AND #11AND #19
