# Supplementary material for: Repetitive transcranial magnetic stimulation for post-stroke depression: An overview of systematic reviews
Source: Front Neurol. 2023 Mar 16;14:930558. doi: 10.3389/fneur.2023.930558 (PMC10061017; doi:10.3389/fneur.2023.930558)
Supplement: Supplementary file 2 [file Table_2.doc]

**Appendix b:** **Common database retrieval strategies**

| **Database** | **Retrieval strategy** |
| --- | --- |
| EMBASE | #1 ‘meta analysis’/ exp OR ‘meta analysis (topic)’/ exp  #2 ‘meta analysis’: ti,ab OR ‘meta analyses’: ti,ab OR ‘meta-analysis’: ti,ab OR ‘meta-analyses’: ti,ab ORmetaanalysis:  Ti,ab OR metanalysis: ti,ab OR ‘met-analysis’: ti, ab OR metaanalyses: ti, ab OR metanalyses: ti,ab OR ‘clinical trial overview’: ti, ab OR ‘clinical trial overviews’: ti,ab  #3 ‘systematic review’/ exp OR ‘systematic review (topic)’/ exp  #4 ‘systematic review’: ti,ab OR ‘systematic reviews’: ti, ab  #5 ‘repetitive transcranial magnetic stimulation’: ti,ab OR ‘transcranial magnetic stimulation’ : ti,ab OR ‘noninvasive brain stimulation’ : ti,ab  #6‘repetitive transcranial magnetic stimulation’/ exp OR ‘transcranial magnetic stimulation’ / exp OR ‘noninvasive brain stimulation’ / exp  #7‘depression’/ exp OR ‘depressed’ / exp OR ‘post-stroke depression’ / exp  #8‘depression’ : ti,ab OR ‘depressed’ : ti,ab OR ‘post-stroke depression’ : ti,ab  #9‘stroke’/ exp OR ‘brain vascular accident’ / exp  #10‘stroke’ : ti,ab OR ‘brain vascular accident’ : ti,ab  (#1 OR #2 OR #3 OR #4) AND (#5 OR #6) AND (#7 OR #8)AND (#9 OR #10) |
| Web of Science | #1 TS =“meta analysis” OR TS =“meta analyses” OR TS =“meta-analysis” OR TS =“meta-analyses” OR TS = “meta-  Analysis” OR TS =“metanalysis”OR TS =“ metaanalyses”OR TS =“systematic review” OR TS =“systematic reviews” OR TS =“clinical trial overview” OR TS =“clinical trial overviews”  #2 TS =“repetitive transcranial magnetic stimulation” OR TS =“transcranial magnetic stimulation” OR TS =“noninvasive brain stimulation”  #3 TS =“depression” OR TS =“depressed” OR TS =“post-stroke depression”  #4 TS =“stroke” OR TS =“brain vascular accident””  #1 AND #2 AND #3 AND #4 |
| Cochrane Library | #1 MeSH descriptor: [post-stroke depression] explode all trees  #2 (depressed):ab,ti,kw OR (depression):ab,ti,kw  #3 #1OR#2  #4 (meta analysis):ab,ti,kw OR (meta analyses):ab,ti,kw OR (meta-analysis):ab,ti,kw OR (meta-analyses):ab,ti,kw OR (meta-Analysis):ab,ti,kw OR (metanalysis):ab,ti,kw OR (metaanalyses):ab,ti,kw OR (systematic review):ab,ti,kw OR (systematic reviews):ab,ti,kw OR (clinical trial overview):ab,ti,kw OR (clinical trial overviews):ab,ti,kw  #5 (repetitive transcranial magnetic stimulation):ab,ti,kw OR (transcranial magnetic stimulation):ab,ti,kw OR (noninvasive brain stimulation):ab,ti,kw  #6 (stroke):ab,ti,kw OR (electroacupuncture):ab,ti,kw  #7 #3 AND #4 AND #5 AND #6 |
| 万方 | 检索表达式（中英文扩展&主题词扩展）： 主题:(系统评价+meta分析+荟萃分析+元分析)*主题:(经颅磁刺激+重复经颅磁刺激+无创性脑刺激)*主题:(抑郁症 +脑卒中后抑郁+中风后抑郁)*主题:(中风+卒中+脑血管意外) |
| CNKI | ( ( (主题=系统评价 或者 题名=系统评价 或者 v_subject=中英文扩展(系统评价) 或者 title=中英文扩展(系统评价)) 或者 (主题=meta分析 或者 题名=meta分析 或者 v_subject=中英文扩展(meta分析) 或者 title=中英文扩展(meta分析)) ) 或者 ( (主题=荟萃分析 或者 题名=荟萃分析 或者 v_subject=中英文扩展(荟萃分析) 或者 title=中英文扩展(荟萃分析)) 或者 (主题=元分析 或者 题名=元分析 或者 v_subject=中英文扩展(元分析) 或者 title=中英文扩展(元分析)) ) ) 并且 ( ( (主题=重复经颅磁刺激 或者 题名=重复经颅磁刺激 或者 v_subject=中英文扩展(重复经颅磁刺激) 或者 title=中英文扩展(重复经颅磁刺激)) 或者 (主题=经颅磁刺激 或者 题名=经颅磁刺激 或者 v_subject=中英文扩展(经颅磁刺激) 或者 title=中英文扩展(经颅磁刺激)) ) 或者 ( (主题=无创性脑刺激 或者 题名=无创性脑刺激 或者 v_subject=中英文扩展(无创性脑刺激) 或者 title=中英文扩展(无创性脑刺激)) ) ) 并且 ( ( (主题=抑郁症 或者 题名=抑郁症 或者 v_subject=中英文扩展(抑郁症) 或者 title=中英文扩展(抑郁症)) 或者 (主题=脑卒中后抑郁 或者 题名=脑卒中后抑郁 或者 v_subject=中英文扩展(脑卒中后抑郁) 或者 title=中英文扩展(脑卒中后抑郁)) ) 或者 ( (主题=中风后抑郁 或者 题名=中风后抑郁 或者 v_subject=中英文扩展(中风后抑郁) 或者 title=中英文扩展(中风后抑郁)) 并且 ( ( (主题=中风 或者 题名=中风 或者 v_subject=中英文扩展(中风) 或者 title=中英文扩展(中风)或者 (主题=卒中 或者 题名=卒中 或者 v_subject=中英文扩展(卒中) 或者 title=中英文扩展(卒中)) 或者 ( (主题=脑血管意外 或者 题名=脑血管意外 或者 v_subject=中英文扩展(脑血管意外) )) (模糊匹配) |
| CBM | #1 "抑郁症"[不加权:扩展]  #2 脑卒中后抑郁  #3 中风后抑郁  #4 (#3) OR (#2) OR (#1)  #5 经颅磁刺激  #6 重复经颅磁刺激  #7 无创性脑刺激  #8 (#7) OR (#6) OR (#5)  #9 系统评价  #10 meta分析  #11 荟萃分析  #12 元分析  #15 (#12) OR (#11) OR (#10) OR (#9)  #16 中风  #17 卒中  #18 脑血管意外  #19 (#18) OR (#17) OR (#16)  #20 (#19) AND (#15) AND (#8) |
| 维普 | ((((((((((((((题名或关键词=系统评价 OR 题名或关键词=evaluation of system) OR 题名或关键词=system assessment) OR 题名或关键词=system evaluation) OR 题名或关键词=systematic assessment) OR 题名或关键词=systematic evaluation) OR 题名或关键词=systematic review) OR 题名或关键词=systematical review) OR 题名或关键词=系统综述) OR 题名或关键词=meta) OR 题名或关键词=荟萃分析) OR 题名或关键词=元分析) OR 题名或关键词=meta分析) AND (((题名或关键词=经颅磁刺激 OR 题名或关键词=重复经颅磁刺激) OR 题名或关键词=无创性脑刺激) )) AND (((题名或关键词=抑郁症 OR 题名或关键词=卒中后抑郁) OR 题名或关键词=中风后抑郁)))AND(((题名或关键词=中风 OR 题名或关键词=卒中) OR 题名或关键词=脑血管意外))) |
